# PDE expression and contractility of seminiferous tubules of the human testis

**DOI:** 10.1186/1471-2210-11-S1-P23

**Published:** 2011-08-01

**Authors:** Caroline Feuerstacke, Sabine Tasch, Johanna Volkmann, Dieter Müller, Ralf Middendorff

**Affiliations:** 1 Department of Anatomy and Cell Biology, Justus Liebig University Giessen, Giessen, Germany

## Background

In the human testis, myofibroblasts are the main cellular components of the lamina propria of seminiferous tubules. These cells are crucial for the transport of immature sperm towards the epididymis. In many cases of disturbed spermatogenesis, the peritubular lamina propria is considerably thickened, with an increase of extracellular components, resembling fibrotic changes in other organs [1]. Myofibroblast dedifferentiation was described in elderly men.

The second messenger cGMP contributes to the regulation of contractile cell function in the testis [2,3]. Sildenafil, an inhibitor of cGMP-hydrolyzing PDE5, is used in an increasing number of young patients to treat pulmonary hypertension. However, there is only scarce knowledge on PDEs in the testis and on possible effects of PDE inhibition in male reproductive organs.

## Materials and results

RT PCR analyses revealed expression of all 22 PDE isoforms in the human testis. Expression pattern of PDEs within the lamina propria was characterized using laser capture microdissection (LCM) followed by RT-PCR analyses. Additional RT-PCR with primer pairs for markers of smooth muscle cells (α smooth muscle actin), Sertoli cells (anti-Müller hormone) and germ cells (CatSperI), could exclude contamination with adjacent tissue during microdissection.

PDE5A, PDE3B, PDE9A and PDE10A were found in the regular lamina propria. These PDEs were also detectable in the thickened (fibrotic) lamina propria, suggesting that loss of these enzymes might not contribute to disturbed spermatogenesis associated with thickened lamina propria. By use of the above mentioned combination of primer pairs and additional testis biopsies displaying the absence of germ cells (Sertoli-cell-only syndrome) PDE5 expression in germ cells could also be shown unequivocally. The dual substrate PDEs PDE2A, PDE3A and PDE11A were only detected in intratubular cells of seminiferous tubules, but were absent from the isolated lamina propria.

Newly developed collagen gel-based assays which analyzed image stacks every 0.1 till 4 sec monitored by a confocal microscope allowed to visualize spontaneous contractions of isolated seminiferous tubules. First data with the PDE5 inhibitor Sildenafil showed a decrease of amplitude and frequency, providing strong evidence for an essential contribution of PDE5 to regulation of tubular contractility. Spontaneous contractility of seminiferous tubules from rats after long-term treatment with sildenafil, however, was conserved.

## Conclusion

The specific expression profile of PDEs in the lamina propria of the human testis and effects of PDE5 inhibition on contractility in isolated seminiferous tubules point to a well orchestrated functional interplay of PDEs for sperm transport within the testis.
